# High Power Distance Enhances Employees' Preference for Likable Managers: A Resource Dependency Perspective

**DOI:** 10.3389/fpsyg.2016.02066

**Published:** 2017-01-09

**Authors:** Cong Wei, Xiaomin Sun, Jia Liu, Chunfang Zhou, Gang Xue

**Affiliations:** ^1^School of Psychology, Beijing Normal University, Beijing Key Lab of Applied Experimental PsychologyBeijing, China; ^2^School of Education, Ningxia UniversityYinchuan, China; ^3^Department of Learning and Philosophy, Aalborg UniversityAalborg, Denmark; ^4^Department of Public Administration, Chinese Academy of GovernanceBeijing, China

**Keywords:** likability, leadership selection, agreeableness, power distance orientation, resource dependence

## Abstract

Is a manager's likability important from an employee's perspective? Research results in this field are scant and inconsistent. The current study explored employees' response to managers' likability and the moderating effect of power distance at both the cultural and individual levels. In study 1, following the countercultural priming experimental paradigm proposed by Van den Bos et al. (2013), 121 college students from China (a high power distance culture) and 99 college students from Denmark (a low power distance culture) were randomly assigned to either a countercultural (experimental) condition or a control condition. All participants were required to complete a manager selection task using the zero-acquaintance paradigm to measure their preference for likable managers. The results confirmed the moderating role of power distance at the cultural level. Study 2 further explored the moderating effect of power distance orientation at the individual level, as well as the boundary condition of the degree of resource dependence from the employee's perspective. One hundred and three Chinese participants with work experience were randomly assigned to either the subordinate perspective (high resource dependence) or the HR department perspective (low resource dependence) condition and completed the same task as in study 1. The results suggested that high power distance-oriented participants demonstrate stronger preference for likable manager candidates than do low power distance-oriented participants. In addition, these findings hold only when employees expect a high resource dependence relation with the manager. Theoretical and practical implications of the research findings and future research directions were discussed.

## Introduction

What leadership characteristics do subordinates feel are important? This question must be answered during national elections, when CEOs are replaced, and when university presidents retire (Hogan et al., 1994). Competence and likability are the two most important criteria that people consider when choosing their work partners. In regard to choosing leaders, it has long been accepted that competence is crucial, while likability is not essential (Casciaro and Lobo, 2005). In the study entitled “*I'm the Boss! Why Should I Care If You Like Me?,”* Zenger and Folkman (2013) refute the common misconception that leaders can be highly effective without being likable. In a study of 51,836 leaders, they found just 27 who were rated at the bottom quartile in terms of likability but in the top quartile in terms of overall leadership effectiveness.

Likability can be defined as a pleasant, nice, and agreeable personality (Hogan et al., 1994). The role of a candidate's likability in leadership selection has long been overlooked (Furnham, 2002). The current research focuses on examining how employees' preference for manager candidates is affected by these candidates' likability. Do employees prefer a manager with greater likability, other things being equal?

Answers to this question require knowledge on bottom-up perception and evaluation. However, very few studies have been conducted in this area. A lot of scientific researches have been conducted on the top-down selection process, which often involve specifying qualities, traits, and skills (or competencies) that ideal candidates should possess. This criterion reflects what leaders want for their subordinates (Anderson and Cunningham-Snell, 2000). However, when people evaluate manager candidates from a bottom-up perspective, they might look for different qualities and make different choices (Cook and Emler, 1999).

Cook and Emler (1999) found that from the bottom-up perspective, employees paid more attention to moral vs. technical credentials. That is, employees tend to compromise morality for competence when choosing their managers, while those utilizing the top-down perspective do the reverse. Furnham et al. (2012) examined the way people weigh information when making upward decisions regarding who they would like as a boss. In their study, participants rank ordered 16 potential bosses that differentiated between the sex, age, intelligence (IQ) and emotional intelligence (EQ) scores of possible candidates. The results showed a strong preference for high EQ and IQ, with EQ being more powerful that IQ. These findings give us some preliminary evidence suggesting that unlike in top-down selection, in the bottom-up selection process, manager candidates' likability might be much more valued by subordinates.

However, some related literature fails to support the above argument. Since likability is closely related to agreeableness in the five-factor model of personality, how managers' likability influences employees' preference is theoretically related to the leader trait paradigm, which focuses on the relation between leadership and factors in the five-factor model of personality (Zaccaro et al., 2004; Judge et al., 2009). Surprisingly, in the most comprehensive meta-analysis of the leader trait paradigm to date, Judge et al. (2002) found that agreeableness showed a relatively weak correlation with leadership (ρ = 0.08). Especially when focusing on the leadership emergence criterion, the relation between agreeableness and leadership was even weaker (ρ = 0.05).

The inconsistencies in the extant literature suggest that some moderating factors may influence employees' preference for a manager's likability. The current study focuses on the potential moderating role of power distance. The work relationship between managers and their subordinates is dependent on the power distance of the culture (Białas, 2009), which is defined by Hofstede as “…the extent to which the less powerful members of organizations and institutions accept and expect that power is distributed unequally” (Hofstede and Hofstede, 2001). In particular, the construct tends to be identified with the willingness of the less powerful members of society to accept their lower status and authority roles vis-a-vis the more powerful members (Adler, 1991). The current research aims to explore how employees' responses to managers' likability are shaped by the power distance of the society and personally held power distance beliefs.

It is important to understand the bottom-up selection process because a high-quality relationship between leaders and subordinates is based on mutual liking, trust, and influence (Bernerth et al., 2007), which, in turn, has been linked to improved outcomes, such as organizational citizenship behavior, job performance, organizational commitment, and general job satisfaction (Dulebohn et al., 2012). The results of the current research could enrich the literature on bottom-up selection and evaluation and shed light on the leader trait paradigm by providing a possible avenue to reconcile the inconsistent research findings in a coherent fashion.

## Theoretical development

### The double-sidedness of managers' likability

Judge et al. (2009) concluded that agreeableness, which is closely related to likability, has both bright and dark sides as far as leadership emergence and leadership effectiveness are concerned. On its bright side, for example Costa and MacCrae (1992) suggested that agreeable individuals tend to be regarded as trusting and trustworthy because of their modesty and altruistic behavioral styles. Trusting and trustworthy individuals are more likely to be recognized as leaders because these characteristics are critical for constructing and maintaining a good relationship between the leader and follower. On the dark side of agreeableness, for example, Graziano et al. (1996) found that leaders with high agreeableness tend to avoid interpersonal conflict and that the personal feelings and desires of others at work may occasionally have too much influence on their decisions. These characteristics therefore harm their leadership and decisiveness. Thus, the weak relationship found between likability and agreeableness in the meta-analysis by Judge et al. (2002) might be due to the dual-edged characteristic of the construct.

The double-sidedness of a manager's likability can be better understood within the warmth-competence social cognition framework. Research has established that perceived warmth and competence are the two universal dimensions of human social cognition (Fiske et al., 2007). The warmth dimension assesses the other's perceived intent in the social context, including friendliness, trustworthiness, and morality, whereas the competence dimension relates to the perceived capability to enact intent, including intelligence, skill, and efficacy. There are persistent findings suggesting that people tend to see warmth and competence as inversely related. If there is an apparent surplus of one trait, they infer a deficit of the other (Judd et al., 2005; Cuddy et al., 2008). Since likability clearly falls into the warmth dimension, the double-sidedness of managers' likability is exactly a reflection of the relationship between warmth and competence.

### The primacy of warmth and the moderators of the likability-preference effect

Although both warmth and competence are fundamental to social perception, considerable evidence suggests that warmth judgments are primary: warmth is judged before competence, and warmth judgments carry more weight in affective and behavioral reactions (Wojciszke and Abele, 2008; Cuddy et al., 2009). For example, children as young as three use warmth judgments before competence judgments to make decisions about new people they encounter (Mascaro and Sperber, 2009). These warmth primacy effects can be explained by the importance of first assessing others' intentions (whether they are friends or foes) before determining their ability to carry out those intentions.

However, a puzzle remains. Given that likability has both a bright side (warmth, good intentions) and a dark side (less competence than expected), the primacy of warmth in social perception likely predicts that employees demonstrate a stronger preference for more likable managers. Why is there only a very weak positive relationship between agreeableness and leadership emergence, according to Judge et al. (2002)'s meta-analysis?

Researchers suggested that the priority of detecting warmth over competence, although robust, is moderated by perceivers and situations. For example, women's preference for others' warmth is stronger than men's because warmth traits affect women's lives more, whereas competence traits affect men more (Abele, 2003). Additionally, individual with collectivistic orientations demonstrate a stronger preference for warmth because they emphasize this social dimension, whereas individualistic orientations emphasize the competence dimension (Wojciszke, 1997).

Cuddy et al. (2008) proposed that the primacy of warmth is also moderated by self-other outcome dependency. For example, Casciaro and Lobo (2005) found that when individuals choose work partners, warmth is more important than competence. However, when managers choose their subordinates, competence is deemed more important than the interpersonal dimension of job performance (warmth) (Wojciszke and Abele, 2008). Cuddy et al. (2008) argued that competence affects global evaluations of others when it contributes to the perceiver's well-being, such as when an employee's positive outcome is contingent on his or her manager's competence, the employee's preference toward the manager's likability might become weaker or even reverse. On the contrary, when an employee believes that the manager's likability rather than competence greatly determines his or her benefit, the employee will choose a highly likable manager without a doubt.

To better understand the complex calculus of employees regarding the relative weight of the two sides of managers' likability, it is necessary to look into the process of bottom-up selection from employees' perspective since impression formation is part of the selection process and frequently a motivated process (Fiske and Neuberg, 1990). Cook and Emler (1999) proposed that impression formation is motivated by the anticipation of a relationship with the target of perception, and the perceiver's motivation is therefore a function of the kind of relationship anticipated. Consistent with this view, in the case of bottom-up selections, the anticipated relationship involves a power differential, and perceivers normally anticipate having less power than the person being perceived. This context is quite different from top-down selections, in which perceivers usually have more power than the target.

When people hold positions of power, their judgments, decisions, and actions are more consequential for the welfare, rights and interests of other persons than those of people who lack such power. In terms of their expected dependence on the relationship, leaders, or supervisors differ from those who are led or who are supervised. Additionally, employees' perception of their dependence on the relationship may differ from one another. One important determinant of this dependence perception is the power distance of the society within which the relationship exists.

### Power distance and the resource dependency perspective

The cultural context plays an important role in the decision making process. In societies with high power distance, the superior more often makes decisions without the subordinates' participation. Both managers and subordinates consider each other to be existentially unequal. People accept the inequalities of power and need no further justification. In contrast, in societies with low power distance, superiors and subordinates are perceived as partners. Employees consider that they have rights to participate in making decisions that concern them (Sagie and Aycan, 2003). People strive to equalize the distribution of power and demand justification for inequalities of power. Competence is used to acquire expert power rather than to signal social status (Mead, 2003). Moreover, in high power distance cultures, there is a fear of punishment in cases of disagreement with the management's decision. This fear is weaker in low power distance cultures (Mead, 2003).

From the resource dependency perspective (Emerson, 1962), followers often find it advantageous to maintain a good relationship with their leaders because the leaders control their access to valued resources (Aquino et al., 2006). This is particularly true for people in societies exhibiting a large degree of power distance because they accept and expect inequalities between powerful and less powerful people. Consequently, the degree of followers' dependence on their leaders is high, and they are more inclined to prefer a leader with high likability in order to retain access to valued resources.

With regard to the likability-competence tradeoff that employees face when choosing their managers, for employees in societies with a small degree of power distance, they strive to justify the difference between high and low power. Competences are reasonable evidence that a candidate is qualified to hold a higher-power position. Thus, competence is relatively more important for employees in low power distance cultures than for those in high power distance cultures. Accordingly, the preference for likable managers might be weaker. However, the picture differs for employees in societies with a large degree of power distance, where people routinely accept the authority of power holders without any doubt. For these employees, the most salient and urgent issue is not power justification (i.e., who is qualified to be a manager), but rather, their personal likelihood of accessing the valued resources they desire. The strong resource dependence relation that exists between the manager and his or her subordinate motivates the latter to choose likable candidates in order to maintain a good relationship with managers so that they have a greater likelihood to obtain what they want. It is rational and beneficial for them to prefer likability to competence when choosing their managers. Thus, we propose the following hypothesis:

**Hypothesis 1**. Employees in high power distance cultures demonstrate a stronger preference toward likable managers than employees in low power distance cultures.

### Power distance orientation

Recent meta-analyses about leadership and employees' attitudes (Dulebohn et al., 2012; Rockstuhl et al., 2012) argue that individual-level variation in cultural values exists and can be larger than country-level variation. Thus, we can further confirm the moderating role of power distance at the individual level.

To distinguish between power distance at the country and individual levels of analysis, power distance orientation was used to indicate an individual-level construct (Kirkman et al., 2009). Power distance orientation refers to the extent to which people accept unequally distributed power in a society or in an organization (Hofstede, 1984). Employees with a high power distance orientation accept status differences and are willing to comply with decisions made by powerful others (Chen and Aryee, 2007; Farh et al., 2007). Thus, high power distance-oriented employees tend to perceive that they are heavily dependent on their managers; in other words, the distribution of valuable resources depends to a large degree on their relationship with their managers. A likable manager who tends to have good intentions toward them reassures them when they are given the chance to select their managers. Thus, we propose the following hypothesis:

**Hypothesis 2**. Employees with high power distance orientation demonstrate a stronger preference for likable managers than employees with low power distance orientation.

Apart from the individual differences in power distance orientation, employees' preference for managers' likability is also shaped by the structural relationship between the perceiver and the perceived target. As previously discussed, because power resides implicitly in others' dependence, the degree of dependence might be one of the most important contextual factors that shape employees' expectations of their managers. As employees feel more strongly that they are dependent on their managers, they become more motivated to maintain a good relationship with the leader, and thus, they are more likely to choose a likable manager. If employees do not believe that they have any dependence on their managers, they do not need to choose likable managers who might be deficient in their capabilities. Thus, we propose a boundary condition of employees' preference for a likable manager:

**Hypothesis 3**. High power distance-oriented employees' preference for likable managers is stronger than that of low power distance-oriented employees in high dependence relations. However, when the degree of dependence is low, high power distance-oriented employees may show neither greater nor lower preference for likable managers than low power distance-oriented employees.

The current study differentiates two perspectives. One is a high dependence perspective, in which subordinates choose their direct superior. Another is a low dependence perspective, in which employees working in the HR department choose a manager for another department in the organization and have no direct working relationship with that manager. We expected that the moderating role of power distance orientation holds only in the subordinate perspective condition.

### Overview of the current studies

The current research examined the moderating effects of power distance on employees' preference toward managers' likability. In study 1, we explored the effect at the cultural level by introducing an experimental condition wherein a countercultural norm is made salient in both high and low power distance cultures. In study 2, we confirmed our findings from study 1 by exploring the moderating role of power distance orientation at the individual level. Study 2 also explored the degree of resource dependence as a boundary condition for the mechanism. In both studies, to clarify the effect of managers' likability on employees' preferences, we controlled for managers' gender, participants' gender, managers' perceived competence, perceived decisiveness, and perceived leadership, which have some relationship to leadership emergence (Judge et al., 2002).

The project was reviewed and approved by the Academic Ethics Committee of the School of Psychology at Beijing Normal University (approval number: 2015008) before being conducted. All participants gave their written informed consent prior to the experiment and were informed of their right to abort the experiment at any time.

## Study 1

Study 1 tested the hypothesis that power distance at the cultural level moderates the relationship between a manager's likeability and employees' preference following the countercultural priming experimental paradigm proposed by Van den Bos et al. (2013). Van den Bos et al. (2013) argued that participants in cross-culture research cannot be randomly assigned to different cultures. However, it is possible to randomly assign people from different cultures to either a control condition or a countercultural condition. In the control condition, no cultural values are emphasized explicitly; hence, people are likely to default to the values and beliefs that are predominant in their culture. In the experimental condition, “countercultural” psychological states were elicited, that is, low power distance was primed in the high power distance culture and vice versa. To the extent that the results in countercultural conditions meaningfully differ from those observed in the control condition, we gain insight into the psychological dimensions that account for cross-cultural differences in people's reactions.

According to Hofstede and Hofstede (2001), China scores high on the power distance index: 80 points compared to a world average of 56.5. In contrast, Denmark is considered to be a low power distance culture with a power distance index of 18. We expect that in the experimental conditions in both countries, the opposite effect will take place. In Denmark, we expect the experimental condition to show results similar to those found in the control condition in China (employees demonstrate a stronger preference toward likable managers), whereas in the experimental condition in China, we expect results similar to those found in the control condition in Denmark (employees demonstrate weaker or no preference for likable managers).

In this study, we examined employees' perceptions and preferences in a manager selection task using the zero-acquaintance paradigm, in which participants rated unacquainted likable and unlikable manager candidates on a variety of dimensions. The accuracy of judgments in the zero-acquaintance situation has been fairly well established (Ambady et al., 1995).

### Methods

#### Material development

In this study, manager candidates' likability was manipulated by using headshots. Recent evidence has suggested that “observers are able to form reasonably accurate impressions for a number of traits including likability simply on the basis of physical appearance” (Naumann et al., 2009).

Headshots of manager candidates were extracted from a neutral emotion headshot dataset (200 photographs of individuals of Han ethnicity, 100 females, and 100 males, designed by psychology researchers at Weinan Normal University). The photographs in the dataset were in a standardized format and did not have clearly noticeable features, which mitigated possible influence from variation in the photographs' characteristics.

In a pilot study, all the photographs in the dataset were evaluated by an independent sample of 41 respondents (30 females, 5 missing). In accordance with previous work (Geys, 2014), participants were asked: “Based on the picture provided, what do you think of this person—compared with people living in your country—in terms of his/her likability (i.e., how nice, pleasant and agreeable do you find this person)?” using a 5-point scale (1 = “not likable at all,” and 5 = “very likable”). To control for the effect of facial attractiveness, participants were also required to evaluate “how attractive do you think this person is?” using a five-point scale (1 = “not attractive at all,” and 5 = “very attractive”). Overall, 1640 evaluations were obtained with an average of 8.2 evaluations per photograph. Following the “truth of consensus” method (Poutvaara et al., 2009), we calculated the average of the independent evaluations across the raters for every photograph.

Based on the average evaluation scores on likability and facial attractiveness for each photograph, 16 photographs were selected, including 8 female photographs with high and low likability (for high-likability photographs, *Ns* = 30, *M* = 2.633, *SD* = 1.066; for low-likability photographs, *Ns* = 16, *M* = 1.688, *SD* = 0.793; *p* = 0.002) and 8 male photographs with high and low likability (for high-likability photographs, *Ns* = 30, *M* = 2.600, *SD* = 1.102; for low-likability photographs, *Ns* = 30, *M* = 1.767, *SD* = 0.679; *p* = 0.001). The candidates' attractiveness was controlled by keeping the photographs within one standard deviation on the facial attractiveness evaluation obtained from the same survey (average ratings were 2.813 and 2.533 for unlikable and likable females, respectively, *p* = 0.494, and 1.733 and 1.967 for unlikable and likable males, respectively, *p* = 0.319).

Since previous work has shown that individuals agree across cultures on the traits that they infer from faces (Albright et al., 1997; Rule et al., 2010), this pool of headshots was used in later experiments, including those for both Chinese and Danish samples.

#### Participants and design

One hundred twenty-one Chinese college students (68 females) from Beijing Normal University and Beijing Jiaotong University in China and 99 Danish college students (44 females) from Aalborg University in Denmark participated in the study. In each country, participants were randomly assigned to either the experimental condition or the control condition. Each participant received a small gift for his or her participation.

#### Experimental procedure

The procedure was similar to that of Van den Bos et al. (2013). Participants were informed at the outset that the stimulus materials would be presented to them in two unrelated parts. In the first part of the study, participants completed a two-question exercise designed to prime or not prime them with countercultural values regarding power distance. Specifically, low power distance was made salient in China; whereas high power distance was made salient in Denmark. In the control conditions in both countries, power distance was not primed.

Following Van den Bos et al. (2013), the instructions in the experimental condition in which low power distance was primed among Chinese participants were as follows:

This part of the study will focus on other people's potential to determine or direct your behavior. More specifically, we ask you to read some materials and answer some questions that ask you to imagine that you and those who have power over you (e.g., employers, teachers, your parents, etc.) regard each other as equals. Thus, formal positions do not matter much.

Question 1: Please describe a situation from your own life in which there was small distance between you and the person who formally had power over you. Thus, we ask you to imagine and describe a situation in which you and a person who formally had power over you regarded each other more or less as equals. Can you briefly describe this situation?

Question 2: Imagine there is small distance between you and a person who has power over you; thus, that you and a person who formally has power over you regard each other more or less as equals. Could you briefly describe how you would feel in such a situation and why it may be a good thing when a person with power treats you as equal?

The instructions in the experimental condition in which high power distance was primed among Danish participants were as follows:

This part of the study will focus on other people's potential to determine or direct your behavior. More specifically, we ask you to read some materials and answer some questions that ask you to imagine that you are the less powerful and are willing to accept that those who have power over you (e.g., employers, politicians, police officers, etc.) have it because of their formal, hierarchical position.

Question 1: Please describe a situation from your own life in which there was large distance between you and the person who formally had power over you. Thus, we ask you to imagine and describe to us a situation in which you were willing to accept that a person had power over you because of the person's formal, hierarchical position. Can you briefly describe this situation?

Question 2: Imagine there is large distance between you and a person who has power over you; thus, that you are willing to accept that a person who formally has power over you because of the person's formal, hierarchical position. Can you briefly describe how you would feel in such a situation and why it may be a good thing when a person with power occupies this powerful position by means of a formal, hierarchical appointment?

In the control condition, no power distance was made salient. Participants in the control condition received the following instructions:

In this part of the study, we will ask you to read some materials and answer some questions concerning watching television. We ask you that you carefully read them and complete them.

Question 1: Please briefly describe the thoughts and emotions that come to mind when you think of the concept of watching TV.

Question 2: Please describe a situation from your own life in which watching TV played a role.

In the second part, following Geys (2014), participants were asked to imagine that they were working in the sales department of a large company. The department was in the process of hiring a new manager. Because the new manager will be their direct superior, their perceptions of and preferences for the candidates would be collected in this task, and their opinions would be seriously considered when the final hiring decision was made (Geys, 2014).

By using a within subject design, each participant was given four manager candidates' resumes to evaluate. Information on the resumes included gender, age, education, work experience, and most importantly, a standardized black-and-white headshot of the candidate. The information in the resumes was designed to be roughly equal, except that the likability of the manager candidates' headshots was manipulated (Geys, 2014). Each participant evaluated 2 resumes with headshots that scored high in likability and 2 resumes with headshots that scored low in likability. All of the headshots were randomly selected from the pre-tested pool of headshots.

After reading the resumes, the participants were asked to evaluate the four candidates on their likability (i.e., how nice, pleasant and agreeable they found the candidates), competence, decisiveness, and leadership using a set of standardized evaluation forms (7-point scale, with 1 being “very negative” and 7 being “very positive”). At the end of the task, they were asked the likelihood that they would choose the candidate as their manager using a 7-point scale (1 was “least possible,” and 7 was “most possible”), keeping in mind that the successful candidate would become their direct manager. To prevent order effects, the headshots selected were assigned to the four resumes randomly, and all participants received resumes in a randomized order. To prevent possible gender effects, the gender of the four candidates to be evaluated was always kept the same (i.e., each participant was presented with either four male or four female candidates).

#### Measures and variables

##### Countercultural power distance manipulation

The countercultural power distance manipulation was dummy-coded with the experimental condition coded as “0” and the control condition coded as “1.”

##### Preference for likable manager candidates

Each participant evaluated 4 manager candidates' resumes: 2 resumes high in likability and 2 low in likability. Each participant's responses on the likelihoods of selecting those 2 likable manager candidates as their direct managers were averaged to represent his or her tendency to select a manager with high likability. Each participant's tendency to select managers with low likability was also computed in the same way. Then, each participant's preference for likable manager candidates was computed by using his or her tendency to select managers with high likability minus that for managers with low likability. Specifically, if the score is significantly greater than 0, the participant demonstrates a likability-preference effect, that is, he or she prefers more likable managers. In contrast, if the score is significantly less than 0, it indicates that the participant demonstrates a likability-aversion effect. Otherwise, if the score shows no significant difference from 0, the participant shows indifference toward managers' likability. This variable was denoted as *likability-preference* in the rest of this study.

In addition to the candidates' likability, the perceived difference in some other aspects of the manager candidates may also affect participants' manager selection preference. The following variables were used as control variables in the statistical process to exclude their effects on participants' preference for likable manager candidates.

##### Perceived difference in high vs. low likable manager candidates' decisiveness

Using the same method used to calculate the *likability-preference* variable, each participant's responses on the perceived decisiveness of those 2 likable manager candidates were averaged to represent his or her perception for likable manager candidates' decisiveness. In the same way, this perception of unlikable manager candidates was also computed. The difference between these two scores form the variable for the perceived difference in high vs. low likable candidates' decisiveness, denoted as δ-perceived-decisiveness in the rest of the study.

##### Perceived difference in high vs. low likable manager candidates' leadership

Computed in the same way as δ-perceived-decisiveness and denoted as δ-perceived-leadership.

##### Perceived difference in high vs. low likable manager candidates' competence

Computed in the same way as δ-perceived-decisiveness and denoted as δ-perceived-competence.

##### Participant gender and candidate gender

The participants' gender and candidates' gender were dummy-coded with female coded as “0” and male coded as “1.”

##### Perceived likability of manager candidates with high vs. low likability

Each participant's responses on the perceived likability of those 2 likable manager candidates were averaged to represent his or her perception of candidates' likability. In the same way, this perception of unlikable manager candidates was also computed. These variables were used in the manipulation check.

### Results

#### Manipulation check

A paired sample *t*-test of perceived likability toward manager candidates regarding high vs. low likability manipulation were conducted in each condition in both the Chinese and Danish samples.

##### Chinese sample

A significant main effect was found in both the experimental condition and the control condition. In the experimental condition, *t*_(56)_ = 9.211, *p* < 0.001. High likability manipulated candidates obtained a significantly higher perceived likability score (*M* = 4.781, *SD* = 0.802) than low likability manipulated candidates (*M* = 3.307, *SD* = 0.875). In the control condition, *t*_(63)_ = 13.050, *p* < 0.001. High likability manipulated candidates obtained a significantly higher perceived likability score (*M* = 4.687, *SD* = 0.974) than low likability manipulated candidates (*M* = 3.055, *SD* = 0.952).

##### Danish sample

A significant main effect was found in both the experimental condition and the control condition. In the experimental condition, *t*_(48)_ = 4.798, *p* < 0.001. High likability manipulated candidates obtained a significantly higher perceived likability score (*M* = 4.398, *SD* = 1.036) than low likability manipulated candidates (*M* = 3.388, *SD* = 1.178). In the control condition, *t*_(49)_ = 5.632, *p* < 0.001. High likability manipulated candidates obtained a significantly higher perceived likability score (*M* = 4.540, *SD* = 1.097) than low likability manipulated candidates (*M* = 3.780, *SD* = 1.135).

These results suggest that the manipulation of manager candidates' likability was successful.

#### Hypothesis testing

Table 1 presents the mean values and standard deviations of the raw data. Table 2 presents the means, standard deviations, and zero-order correlations of variables in study 1.

**Table 1:** **Means, Standard Deviations of raw data in study 1**.

**Samples**	**Variables**	**Experimental Condition**					**Control Condition**				
		***Ns***	**High Likability**		**Low likability**		***Ns***	**High Likability**		**Low likability**	
			***M***	***SD***	***M***	***SD***		***M***	***SD***	***M***	***SD***
Chinese	1. Perceived-Likability	57	4.781	0.802	3.307	0.875	64	4.688	0.974	3.055	0.952
	2. Perceived-Decisiveness	57	4.474	0.671	5.018	0.726	64	4.531	0.930	4.984	0.811
	3. Perceived-Leadership	57	4.737	0.791	4.728	0.780	64	4.727	0.943	4.898	0.822
	4. Perceived-Competence	57	4.816	0.816	4.623	0.781	64	4.734	0.972	4.750	0.870
	5. Likelihood to select candidates as manager	57	4.377	0.983	3.702	1.004	64	4.523	0.870	3.258	0.996
Danish	1. Perceived-Likability	49	4.398	1.036	3.388	1.178	50	4.540	1.097	3.780	1.135
	2. Perceived-Decisiveness	49	4.378	0.938	3.949	1.247	50	4.530	1.104	4.560	1.320
	3. Perceived-Leadership	49	4.510	0.832	4.082	1.292	50	4.660	1.109	4.561	1.257
	4. Perceived-Competence	49	5.000	1.005	4.337	1.456	50	5.000	1.305	4.940	1.384
	5. Likelihood to select candidates as manager	49	4.512	0.933	3.235	1.204	50	4.300	1.000	4.130	1.129

**Table 2:** **Means, Standard Deviations, and Intercorrelations among variables in study 1**.

**Samples**	**Variables**	***Ns***	***M***	***SD***	**1**	**2**	**3**	**4**	**5**
Chinese	1. Candidate gender^a^	121	0.510	0.502					
	2. Participant gender^a^	121	0.440	0.498	0.280				
	3. δ-perceived decisiveness	121	−0.497	0.995	0.046	0.047			
	4. δ-perceived leadership	121	−0.087	0.957	0.206^*^	0.107	0.438^***^		
	5. δ-perceived competence	121	0.083	0.932	0.078	0.038	0.308^***^	0.506^***^	
	6. Likability-preference	121	0.996	1.264	−0.200^*^	−0.050	−0.033	0.029	0.296^***^
Danish	1. Candidate gender^a^	99	0.515	0.502					
	2. Participant gender^a^	99	0.556	0.499	−0.014				
	3. δ-perceived decisiveness	99	0.197	1.131	−0.073	0.021			
	4. δ-perceived leadership	98	0.265	1.225	−0.013	0.049	0.661^***^		
	5. δ-perceived competence	99	0.359	1.088	−0.201^*^	0.005	0.667^***^	0.733^***^	
	6. Likability-preference	99	0.717	1.367	−0.105	−0.134	0.442^***^	0.387^***^	0.527^***^

a Dummy variable (0, male; 1, female).

*** p < 0.001.

* *p < 0.05*.

First, a three-stage hierarchical regression was conducted to check the moderating effect of the power distance manipulation in each sample. The likability-preference variable was treated as the dependent variable. Candidate gender, participant gender, δ-perceived decisiveness, δ-perceived leadership, and δ-perceived competence were treated as control variables. Candidate gender and participant gender were entered in block 1, followed by δ-perceived decisiveness, δ-perceived leadership, and δ-perceived competence in block 2. Then, a countercultural power distance manipulation was added to block 3. Table 3 presents the results of the regression analysis.

**Table 3:** **Results of study 1 hierarchical regression (standardized coefficient)**.

**Independent variables**	**Dependent variable: Likability-preference**					
	**Chinese Sample**			**Danish Sample**		
	**Step 1**	**Step 2**	**Step 3**	**Step 1**	**Step 2**	**Step 3**
Candidate gender^a^	−0.199^*^	−0.21^*^	−0.19^*^	−0.101	0.011	0.057
Participant gender^a^	−0.044	−0.046	−0.057	−0.141	−0.151^+^	−0.124
δ-perceived decisiveness		−0.109	−0.139		0.216	0.183
δ-perceived leadership		−0.068	−0.047		−0.075	−0.036
δ-perceived competence		0.382^***^	0.408^***^		0.447^**^	0.375^**^
Countercultural power distance manipulation			0.259^**^			−0.262^**^
*R^2^*	0.042	0.159	0.224	0.030	0.327	0.386
*ΔR^2^*	0.042	0.117	0.065	0.030	0.297	0.059
*SE of Estimate*	1.248	1.184	1.143	1.365	1.155	1.120
*F for ΔR^2^*	2.590	5.321^**^	9.528^**^	1.470	13.564^***^	8.705^**^
*F*	2.590	4.343^***^	5.475^***^	1.470	8.960^***^	9.542^***^

Ns = 121 for Chinese sample, Ns = 99 for Danish sample.

a Dummy variable (0, male; 1, female). All the contingent independent variables were grounded centered.

*** p < 0.001,

** p < 0.01,

* p < 0.05,

+ *p < 0.1*.

The results based on the Chinese sample suggested, in the first block, that candidate gender emerged as a significant negative predictor, β = −0.199, *p* = 0.029, Δ*R*^2^ = 0.042, *F*_*change*_ (2, 118) = 2.590, *p* = 0.079. After adding δ-perceived decisiveness, δ-perceived leadership, and δ-perceived competence in the second step, δ-perceived competence emerged as a significant positive predictor, β = 0.382, *p* < 0.001, Δ*R*^2^ = 0.117, *F*_*change*_ (3, 115) = 5.321, *p* = 0.002. The effect of candidate gender was still significant. After adding a countercultural power distance manipulation in the third step, the low power distance manipulation emerged as a significant positive predictor, β = 0.259, *p* = 0.003, Δ*R*^2^ = 0.065, *F*_*change*_ (1, 114) = 9.528, *p* = 0.003. The effects of candidate gender and δ-perceived competence were still significant. The results suggested that the likability-preference effect was moderated by the low power distance manipulation. Specifically, participants who were primed with low power distance in the experimental condition (*M* = 0.693, *SD* = 1.352) show a lower preference for likeable managers than those in the control condition (*M* = 1.123, *SD* = 1.262).

Results based on the Danish sample suggest that in the first block, none of gender variables emerged as significant predictors, Δ*R*^2^ = 0.030, *F*_*change*_ (2, 95) = 1.470, *p* = 0.235. After adding δ-perceived decisiveness, δ-perceived leadership and δ-perceived competence in the second step, δ-perceived competence emerged as a significant positive predictor, β = 0.447, *p* = 0.002, Δ*R*^2^ = 0.297, *F*_*change*_ (3, 92) = 13.564, *p* < 0.001. After adding a countercultural power distance manipulation in the third step, the high power distance manipulation emerged as a significant negative predictor, β = −0.262, *p* = 0.004, Δ*R*^2^ = 0.059, *F*_*change*_ (1, 91) = 8.705, *p* = 0.004. The effect of δ-perceived competence was still significant. The results suggest that the likability-preference effect was moderated by the high power distance manipulation. Specifically, participants who were primed with high power distance in the experimental condition (*M* = 1.276, *SD* = 1.511) showed a stronger preference toward likeable managers than participants in the control condition (*M* = 0.170, *SD* = 0.940).

To further test whether individuals in high power distance culture demonstrate a significant likability-preference effect and individuals in low power distance culture demonstrate a significant likability-aversion effect, we checked the main effect of likability manipulation in the control condition of each country. A regression analysis was conducted for each control condition. Likability-preference was treated as the dependent variable. Candidate gender, participant gender, δ-perceived decisiveness, δ-perceived leadership, and δ-perceived competence were treated as control variables. An intercept of the regression model significantly greater or less than zero suggests a main effect of the likability manipulation.

For the control condition of the Danish sample, the intercept is not significant, *B* = 0.429, *p* = 0.122, which indicates that participants in Denmark normally show no preference for likable vs. unlikable manager candidates. For the control condition of the Chinese sample, the results of the regression analysis suggest significantly positive main effects of the likability manipulation in the control condition (*B* = 1.601, *p* < 0.001). This suggests that Chinese participants demonstrated a strong likability-preference effect.

Combining the results for both the Chinese and Danish samples, we can see that participants from the high power distance culture showed a likability-preference effect, while participants from the low power distance culture showed neither a likability-preference effect nor a likability-aversion effect. The results also suggested that the likability-preference effect can be weakened by low power distance priming in a high power distance culture and strengthened by high power distance priming in a low power distance culture. Thus, Hypothesis 1 was supported.

## Study 2

Study 2 further explored the moderating effect of power distance orientation at the individual level and the degree of resource dependence as a boundary condition for the moderating effect of power distance orientation.

### Methods

#### Participants and procedure

One-hundred and fourteen Master of Applied Psychology students (64 females) at Beijing Normal University were recruited and randomly assigned to either the subordinate perspective or the HR department perspective condition. All the students had full-time jobs in various types of organizations. They completed their courses mostly during weekends. Participants received 1 course credit for their participation.

In total, 59 participants (31 females) were assigned to the subordinate condition, and 55 participants (33 females) were assigned to the HR condition. Data on 11 individuals (6 in the subordinate condition and 5 in the HR condition) were excluded from the final data analysis because they failed to sign the consent form.

In the subordinate condition, participants were required to complete the same manager selection task as in study 1. In the HR condition, instead of being asked to choose their direct superior, participants were asked to imagine that they were employees in the organization's human resources department and that they would not personally be working with the to-be-recruited manager.

Perceived likability, perceived competence, perceived decisiveness, perceived leadership, and the likelihood that they would select the candidates as managers were measured in the same way as in study 1. Additionally, each participant's power distance orientation was collected at the end of the measures; 80 participants (71 valid data) were required to complete a collectivism-individualism scale.

#### Measures and variables

##### Resource dependence relation manipulation

Resource dependence relation conditions were dummy-coded. The subordinate condition was coded as “0,” and the HR condition was coded as “1.”

##### Power distance orientation

Power distance orientation was measured by an 8-item scale developed by Earley and Erez (1997) using a 5-point scale (1 was “totally disagree,” and 5 was “totally agree”). This is a widely used tool for power distance orientation measurement. According to previous research, the Cronbach's alpha of the scale ranged from 0.65 to 0.71 (Kirkman et al., 2009). In the current study, the Cronbach's alpha of the 8-item scale was 0.644. Further confirmatory factor analysis suggested that the item loading of item 2, item 3, and item 8 is below 0.30. After deleting these 3 items, the Cronbach's alpha of the resulting 5-item scale turned to be 0.731. Thus, the 5-item scale was used in later data analysis (for the confirmatory factor analysis result of the 8-item and 5-item scale, see Appendix A).

##### Individualism/collectivism

Individualism/collectivism was measured by a 6-item scale developed by Srite and Karahanna (2006) using a 5-point scale (1 was “totally disagree,” and 5 was “totally agree”). The Cronbach's alpha of the scale was 0.720 in the current study.

##### Participant gender and candidate gender

Participant gender and candidate gender were dummy-coded, with female coded as “0” and male coded as “1.”

The computation of perception and preference variables toward manager candidates were the same as in study 1, including employees' preference for likable manager candidates, the perceived difference in high vs. low likable manager candidates' decisiveness, leadership, and competence, and perceived likability of manager candidates with high vs. low likability.

### Results

#### Manipulation check

A paired sample *t*-test of perceived likability of manager candidates with high vs. low likability was conducted in each dependence condition. In the subordinate condition, *t*_(52)_ = 8.179, *p* < 0.001, which suggested that high likability manipulated candidates obtained a significantly higher score (*M* = 4.132, *SD* = 0.936) than low likability manipulated candidates (*M* = 2.991, *SD* = 0.874). In the HR condition, *t*_(49)_ = 9.314, *p* < 0.001, which suggested that high likability manipulated candidates obtained a significantly higher score (*M* = 4.250, *SD* = 0.949) than low likability manipulated candidates (*M* = 3.000, *SD* = 0.728). The results suggest that likability manipulations were successful in both conditions.

#### Hypothesis testing

Table 4 presents the mean values and standard deviations of raw data. Table 5 presents the means, standard deviations, and zero-order correlations of variables in study 2.

**Table 4:** **Means, Standard Deviations of raw data in study 2**.

**Variables**	**Subordinate Condition**					**HR Condition**				
	***Ns***	**High Likability**		**Low likability**		***Ns***	**High Likability**		**Low likability**	
		***M***	***SD***	***M***	***SD***		***M***	***SD***	***M***	***SD***
1. Perceived-Likability	53	4.132	0.936	2.991	0.874	50	4.250	0.949	3.000	0.728
2. Perceived-Decisiveness	53	4.472	0.857	4.726	0.655	50	4.380	0.786	4.880	1.028
3. Perceived-Leadership	53	4.509	0.858	4.443	0.738	50	4.590	0.819	4.620	0.972
4. Perceived-Competence	53	4.736	0.939	4.557	0.964	50	4.620	0.855	4.560	0.946
5. Likelihood to select candidates as manager	53	4.349	0.928	3.566	1.065	50	4.120	0.982	3.740	1.101

**Table 5:** **Means, Standard Deviations, and Intercorrelations among variables in study 2**.

**Conditions**	**Variables**	***Ns***	***M***	***SD***	***1***	***2***	***3***	***4***	***5***	***6***
Subordinate	1. Candidate gender^a^	53	0.510	0.505						
	2. Participant gender^a^	53	0.43	0.500	−0.131					
	3. δ-perceived decisiveness	53	−0.255	0.757	0.170	−0.033				
	4. δ-perceived leadership	53	0.066	0.844	0.123	−0.115	0.576^***^			
	5. δ-perceived competence	53	0.179	0.951	−0.094	−0.066	0.485^***^	0.680^***^		
	6. Likability-preference	53	0.783	1.364	−0.297^*^	0.014	−0.031	0.347^*^	0.212	
	7. Power distance orientation	53	3.419	0.889	−0.048	−0.036	−0.070	−0.127	−0.154	0.253^+^
HR	1. Candidate gender^a^	50	0.440	0.501						
	2. Participant gender^a^	50	0.460	0.503	0.152					
	3. δ-perceived decisiveness	50	−0.500	1.035	−0.157	−0.117				
	4. δ-perceived leadership	50	−0.030	0.842	−0.089	0.130	0.592^**^			
	5. δ-perceived competence	50	0.060	0.956	−0.226	0.111	0.314^*^	0.611^***^		
	6. Likability-preference	50	0.380	1.043	−0.112	0.204	0.591^***^	0.647^***^	0.427^**^	
	7. Power distance orientation	50	3.828	0.567	−0.116	0.268^+^	0.129	0.173	0.091	0.002

a Dummy variable (0, male; 1, female).

*** p < 0.001,

** p < 0.01,

* p < 0.05,

+ *p < 0.1*.

A three-stage hierarchical regression analysis was performed to investigate the moderating effect of power distance orientation, the dependent relation perspective and the interaction of these two variables. In the hierarchical regression model, likability-preference was treat as the dependent variable. Candidate gender, participant gender, δ-perceived decisiveness, δ-perceived leadership, and δ-perceived competence were treated as control variables. Candidate gender and participant gender were entered in block 1, followed by δ-perceived decisiveness, δ-perceived leadership and δ-perceived competence in block 2. Then, the dependent relation perspective manipulation, power distance and their interaction was added to block 3. Table 6 presents the results.

**Table 6:** **Results of study 2 hierarchical regression for all data (standardized coefficient)**.

**Independent variable**	**Dependent variable: Likability-preference**		
	**Step 1**	**Step 2**	**Step 3**
Candidate gender^a^	−0.203^*^	−0.226^*^	−0.237^**^
Participant gender^a^	0.088	0.090	0.118
δ-perceived decisiveness		0.021	0.001
δ-perceived leadership		0.508^***^	0.539^***^
δ-perceived competence		−0.068	−0.060
Role perspective			−0.167^+^
Power distance orientation			0.290^**^
Interaction: Role perspective* Power distance orientation			−0.257^*^
*R^2^*	0.049	0.278	0.367
*ΔR^2^*	0.049	0.229	0.089
*SE of Estimate*	1.211	1.071	1.019
*F for ΔR^2^*	2.579^+^	10.251^***^	4.423^**^
*F*	2.579^+^	7.468^***^	6.820^***^

Ns = 103.

a Dummy variable (0, male; 1, female). All the contingent independent variables were grounded centered.

*** p < 0.001,

** p < 0.01,

* p < 0.05,

+ *p < 0.1*.

In the first block, candidate gender emerged as a significant negative predictor, β = −0.499, *p* = 0.039, Δ*R*^2^ = 0.049, *F*_*change*_ (2, 100) = 2.579, *p* = 0.081. After adding δ-perceived decisiveness, δ-perceived leadership and δ-perceived competence in the second step, δ-perceived leadership emerged as a significantly positive predictor, β = 0.508, *p* < 0.001, Δ*R*^2^ = 0.229, *F*_*change*_ (3, 97) = 10.251, *p* < 0.001. The effect of candidate gender was still significant. After adding the dependent relation perspective manipulation, power distance orientation and the interaction of these variables in the third step, the dependent relation perspective manipulation emerged as a marginal significant negative predictor, β = −0.167, *p* = 0.058, power distance orientation emerged as a significant positive predictor, β = 0.290, *p* = 0.005, and most importantly, the interaction of the power distance and dependent relation perspective manipulation emerged as a significant negative predictor, β = −0.257, *p* = 0.014. In stage three, Δ*R*^2^ = 0.089, *F*_*change*_ (3, 94) = 4.423, *p* = 0.006.

First, the intercept of likability-preference is significantly larger than zero (*B* = 0.752, *p* < 0.001) after controlling for candidate gender, participant gender, δ-perceived decisiveness, δ-perceived leadership, and δ-perceived competence in stage two. This suggests that employees prefer likable managers, given other things that were equal. The likability-preference effect we found in the Chinese sample in study 1 was replicated.

Second, the significant main effect of power distance orientation and its significant interaction with dependent relation perspective suggested the moderating role of power distance orientation and the boundary condition of dependent relation perspective. That is, in the subordinates' perspective condition, people with a high power distance orientation prefer likable leaders more often than people with a low power distance orientation. However, the interaction term indicates that the same results does not hold in the HR condition. Therefore, the moderation effect of power distance orientation exists only when employees judge from a high dependent relation perspective. While, in the low dependent relation condition, people with a high vs. low power distance orientation show no significant difference in their likability-preference. Hypothesis 2 and 3 were supported.

Finally, we also conducted a three-stage hierarchical regression to test the possible moderating effect of participants' individualism/collectivism. In the hierarchical regression, likability-preference was treated as the dependent variable, and candidate gender and participant gender were entered in block 1, followed by δ-perceived decisiveness, δ-perceived leadership, and δ-perceived competence in block 2. Then, individualism/collectivism was added in block 3, following block 2. In total, there were 33 participants in the subordinate condition and 38 participants in the HR condition. The results suggest that the effect of individualism/collectivism was not significant in the subordinate condition, β = −0.032, *p* = 0.864, Δ*R*^2^ = 0.001, *F*_*change*_ (1, 26) = 0.03, *p* = 0.864, nor in the HR condition, β = 0.028, *p* = 0.838, Δ*R*^2^ = 0.001, *F*_*change*_ (1, 30) = 0.042, *p* = 0.838. Therefore, the likability-preference effect cannot be explained by individual differences in Individualism/Collectivism.

## General discussion

Is a manager's likability important from an employee's perspective? The results of the current research consistently support the idea that employees' attitude toward likable managers depends on the power distance culture in which the employees are situated and the power distance belief the employees hold.

We explore how culture-level differences in power distance affect employees' preference for likable managers. Using a countercultural experimental design, study 1 found that low power distance priming significantly weakened the preference for likable managers among Chinese participants who live in a high power distance culture. High power distance priming significantly facilitated the likability-preference of Danish participants who live in a low power distance culture. Thus, H1 was supported. We further confirmed the moderating role of power distance by switching to individually held beliefs regarding power distance in study 2. The results suggested that compared with those low power distance-oriented individuals, high power distance-oriented individuals demonstrated a stronger likability-preference effect. Thus, H2 was supported. Study 2 also analyzed the interaction of power distance orientation and the dependent relation perspectives. The results suggested that high power distance-orientated individuals demonstrate a stronger preference for likable managers only when they expected a high power dependence relation with the manager. When they do not think they will be highly dependent on the manager, the high power distance-oriented individuals show no greater or lower preference for a likable manager than low power distance-oriented individuals. Thus, H3 was supported.

Our results exclude collectivism/individualism as a potential factor that might influence the likability-preference effect. Research has suggested that collectivism/individualism serves as a moderator of the primacy of warmth (Wojciszke, 1997). Research has also suggested that power distance orientation and collectivism/individualism are closely related to each other (Ghosh, 2011). Is it possible that the preference-likability effect is due to individual differences in collectivism/individualism? The research results in study 2 reject this explanation.

The current research suggests that participant gender is not a significant predictor of people's preference for a manager candidate's likability. This conclusion is consistent across study 1 and study 2. Based on research in the field of the warmth-competence framework, perceivers' gender would be a moderator of the warmth primacy effect, with women more likely than men to demonstrate a stronger preference for traits that fall into the warmth dimension (Abele, 2003). It is possible that in a general social context, a perceiver's gender serves as a possible predictor of his or her perception and subsequent decision making. However, in a manager selection situation, the influence of a perceiver's gender was overwhelmed by his or her individually held belief toward the social context—in our study, their power distance orientation. Additionally, research generally found no correlation between gender and power distance orientation (Lee et al., 2000).

We found a considerable robust likability-preference effect in China, a high power distance culture. This finding is consistent across study 1 and study 2. This finding completely opposes the results of Geys (2014), who found in Norway that, all else equal, managers with higher perceived likeability are preferred less than managers with lower perceived likeability. This likeability-aversion emerges among male and female respondents, affects male and female managers, and holds for preferences expressed from the perspective of both employees and HR departments. Why? Based on the current research, a possible explanation is cultural difference in power distance. Norway is considered to be a low power distance culture, with a power distance index of 31. Based on our research, we question the cultural generalizability of Geys's (2014) suggestion to managers, which is also the title of the study, “Better not look too nice.”

Compared with Geys's (2014) conclusion, the current research provides a more integrated framework with which to understand employees' attitude toward leadership. Employees' preference for managers is affected not only by their perception (perceived likability of the manager) but also by the culture value prevailing in their country (power distance), their personal beliefs (power distance orientation) and contextual factors (i.e., high vs. low dependence relations). It is a dynamic process rather than a static response.

### Theoretical implications

Recent meta-analytic research on culture and leadership (Dulebohn et al., 2012; Rockstuhl et al., 2012) indicates that national culture and cultural orientations are important for understanding employees' perceptions of leadership and their expected relation with leaders. Specifically, increasing numbers of researchers call for both a move beyond individualism/collectivism as the focal cultural value and more attention to the effects of individual-level cultural value orientations on reactions to leaders (Kirkman et al., 2006). The current research is a response to such calls. The findings that power distance and power distance orientation are consistent predictors of employees' preference for their leaders' likability add to the growing research on culture and leadership at both the national and the individual levels.

The current findings on likability and its effect on bottom-up selection are different from researches on charisma and leadership preference. Charisma is used to describe a subset of leaders whose “personal abilities are capable of having profound and extraordinary effects on followers” (House et al., 1979). According to Conger and Kanungo (1987), one essential behavioral component of charismatic leader is “likableness.” However, it is the shared perspective and idealized vision that make charismatic leader a likable hero worthy of identification and imitation. In addition, Willner (1985) suggests that charismatic leadership is neither personality-based nor contextually determined. In the current study, likability is a pleasant, nice and agreeable personality (Hogan et al., 1994). Thus, our finding on likability and leadership preference is a unique contribution distinct from that of charisma.

### Practical implications

As suggested by Geys (2014), understanding bottom-up work relations hierarchically is critical for follower outcomes and leadership and organizational effectiveness. The results of this research remind practitioners that employees' views may be biased by their perspective and power distance orientation in manager selection situations. Feedback and opinions from subordinates might not be as objective as expected because their preference could be the result of a tradeoff between competence and likability based on their own well-being. When a high dependence relation exists, likability instead of competency will be considered first, especially for individuals with high power distance orientation. Thus, manager selection decisions made based on such opinions may be beneficial to subordinates in the short term but might be risky for organizations in the long term.

The findings also have important implications for international organizations and cross-cultural management. They suggest that both competence and likability should be taken into consideration when choosing managers to improve both acceptance from subordinates and organizational effectiveness.

Cuddy et al. (2011) note that accurately answering questions about the relative leadership benefits of expressing warmth versus competence will obviously require the collection of additional empirical data. The current research is the first to suggest that the power distance of a society and employees' power distance beliefs should be taken into consideration when answering that question. Based on our research, it is more important for managers to show warmth when they are interacting with high power distance-orientated subordinates and when they are working in a society or context with a high power distance culture.

### Limitations and directions for future research

First, the results suggest that perceived competence also emerged as a positive predictor of the likability-preference effect under some conditions, although the results are far from consistent. In study 1, both samples demonstrated this result. However, this finding was not replicated in study 2. It is contrary to our expectation based on previous literature reviews. Since warmth and competence are inversely related, if a likability manipulation positively predicts employees' preference, perceived competence should be a negative predictor. We suspect that this result is due partly to the evaluation frame of the current research. In order to make sure people's responses for likability perception are independent from their responses in other dimensions, participants were asked to report perceived likability first, then perceived competence, perceived decisiveness, and perceived leadership. It is possible that the responses regarding likability have some halo effect on participants' evaluation of other dimensions. The same explanation also applies to the results in study 2, in which perceived leadership also emerged as a positive predictor. Future research could try other solutions to make sure the evaluations of these dimensions are independent from each other.

In the current study, participants were required to report the likelihood of choosing likable vs. unlikable manager candidates on a 7-point scale instead of asking them to rank the available manager candidates according to their preferences, which was the case in Geys (2014). We used a 7-point scale because it enabled us to statistically control the influence of perceived difference on other dimensions. However, an obvious strength of asking participants to rank the candidates is that ranking imposes choices, that is, people have to show their priority by putting different candidates in different ranks. They cannot rank two individuals in the same position. However, scores cannot avoid this problem. This methodological difference might affect the results to some extent. Future research could compare these two methods in one experiment to determine potential influence.

In addition to the resource dependency perspective, dependence on the leader could also manifest itself in terms of emotional dependence. For example, based on the research on employees' attachment styles, insecurely attached individuals, including both counter-dependent and over-dependent attachment styles, view relationships differently (Little et al., 2011). Since over-dependent individuals try to achieve security by minimizing their distance from others, it is possible that they tend to prefer likable manager because the leader is the “resource” him/herself, rather than that the leader has access to valued resource. Future research could explore how attachment styles (secure, counter-dependent, and over-dependent) influence employees' leadership preference.

According to the Leader Trait Emergence Effectiveness model proposed by Judge et al. (2009), the links between leadership emergence and leadership effectiveness are moderated by contextual factors, such as culture. Based on the findings of the current research, we argue that such contextual factors may also moderate the relation between traits and leadership emergence. Judge et al. (2009) conclude that not only agreeableness but also other factors in the Big Five have both bright and dark sides. It is possible that weighing the bright and dark sides of the other personality factors will also demonstrate notable cultural differences. Future research in this area is greatly needed.

## Author contributions

Conceived and designed the experiments: CW, XS, JL, and GX. Programmed the task: CW and JL. Performed the experiments: JL, CW, and CZ. Analyzed the data: CW. Wrote the paper: CW, XS, and JL.

## Funding

This research is funded by the National Natural Science Foundation of China (Grant No. 71101012), the Fundamental Research Funds for the Central Universities, and the State Scholarship Fund from the China Scholarship Council (201406045033).

### Conflict of interest statement

The authors declare that the research was conducted in the absence of any commercial or financial relationships that could be construed as a potential conflict of interest.
