# Supplementary material for: High Power Distance Enhances Employees' Preference for Likable Managers: A Resource Dependency Perspective
Source: Front Psychol. 2017 Jan 9;7:2066. doi: 10.3389/fpsyg.2016.02066 (PMC5221487; doi:10.3389/fpsyg.2016.02066)
Supplement: Supplementary file 1 [file DataSheet1.pdf]

Appendix A. The confirmatory factor analysis results of the 8-item and 5-item power distance orientation scales

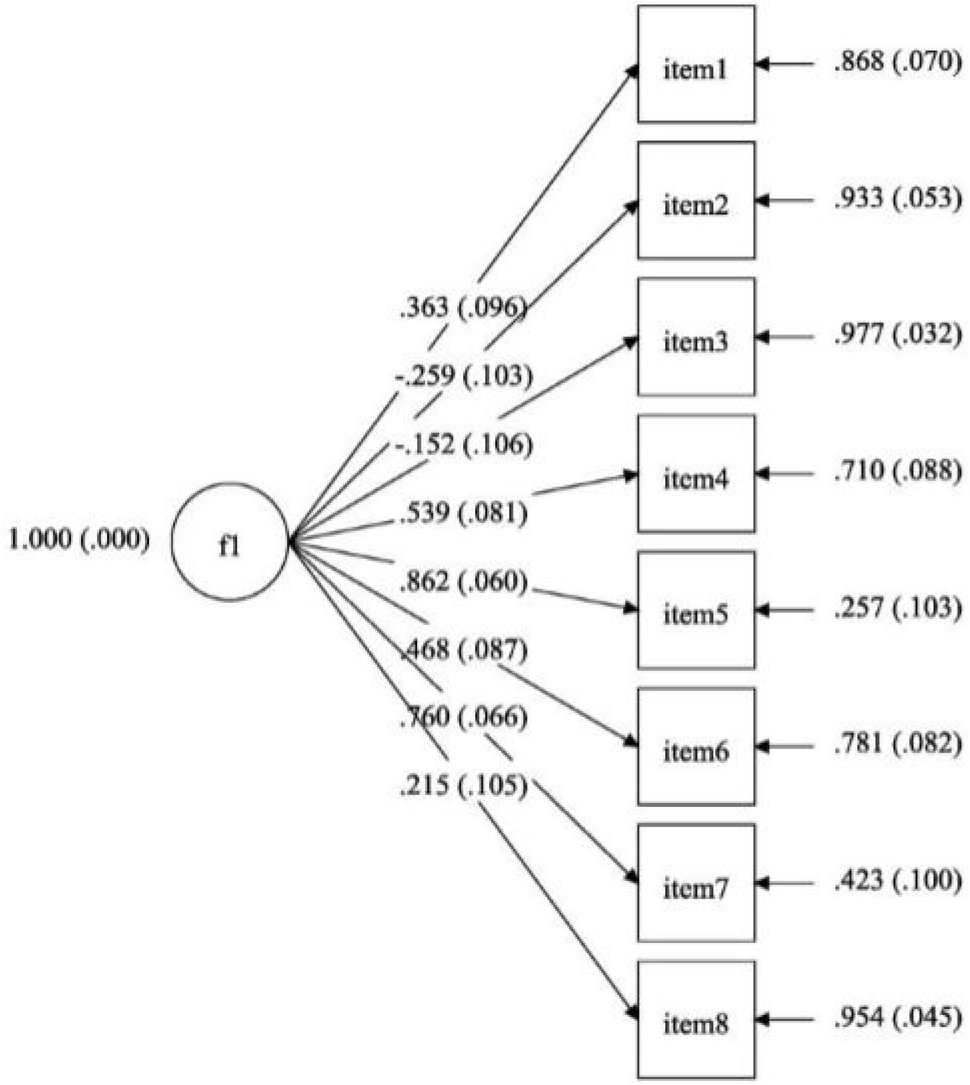

Figure 1. The confirmatory factor analysis results of the 8-item scale

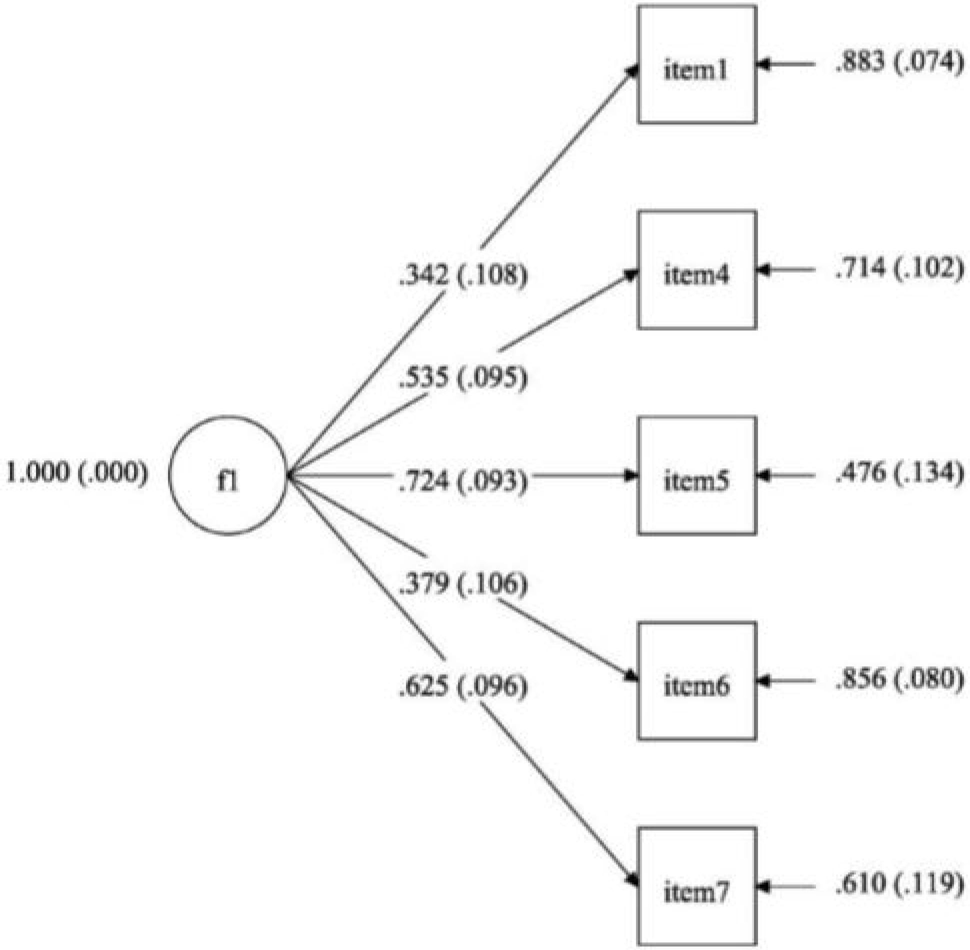

Figure 2. The confirmatory factor analysis results of the 5-item scale
